# The hippocampal FTO-BDNF-TrkB pathway is required for novel object recognition memory reconsolidation in mice

**DOI:** 10.1038/s41398-023-02647-4

**Published:** 2023-11-14

**Authors:** Rui Chang, Shanshan Zhu, Jionghong Peng, Zhenyi Lang, Xinyu Zhou, Hailin Liao, Ju Zou, Peng Zeng, Sijie Tan

**Affiliations:** Department of Histology and Embryology, School of Basic Medicine, Hengyang Medical School, University of South China, Hengyang, China

**Keywords:** Psychiatric disorders, Neuroscience

## Abstract

Memory reconsolidation refers to the process by which the consolidated memory was restored after reactivation (RA). Memory trace becomes labile after reactivation and inhibition of memory reconsolidation may disrupt or update the original memory trace, which provided a new strategy for the treatment of several psychiatric diseases, such as drug addiction and post-traumatic stress disorder. Fat mass and obesity-associated gene (FTO) is a novel demethylase of N6-methyladenosine (m6A) and it has been intensively involved in learning and memory. However, the role of FTO in memory reconsolidation has not been determined. In the present study, the function of FTO in memory reconsolidation was investigated in the novel object recognition (NOR) model in mice. The results showed that RA of NOR memory increased hippocampal FTO expression in a time-dependent manner, while FTO inhibitor meclofenamic acid (MA) injected immediately, but not 6 h after RA disrupted NOR memory reconsolidation. MA downregulated BDNF expression during NOR memory reconsolidation in the hippocampus, while the TrkB agonist 7,8-Dihydroxyflavone (7,8-DHF) reversed the disruptive effects of MA on NOR memory reconsolidation. Furthermore, overexpression of FTO increased BDNF expression via decreasing mRNA m6A in HT22 cells. Taken together, these results indicate that FTO may up-regulate the BDNF-TrkB pathway to promote NOR memory reconsolidation through m6A modification.

## Introduction

Memory is defined as the process of maintaining and subsequent retrieving information over time and could undergo several processes, i.e., acquisition, consolidation, retrieval, reconsolidation. Recent studies found that memory reconsolidation is a crucial stage during which consolidated memories undergo re-stabilization after reactivation (RA) to persist in the brain [1]. Unlike other memory processes, reconsolidation is special because the consolidated memories are labile and susceptible to disruption in a time window of 6 h once reactivated. If the reconsolidation process was disrupted, the original memory trace may be modified, updated, or erased [2]. Based on the theory of memory reconsolidation, novel treatment strategies for psychiatric disorders with maladaptive memories, such as post-traumatic stress disorder (PTSD) [3], anxiety, and addiction [4], are being developed and promising results have been achieved in preclinical studies. For example, inhibition of PARP-1 during the unstable phase of reconsolidation may enhance the extinction of fear memory [5]. Moreover, inhibition of memory reconsolidation also decreased the cue-activated drug-seeking behavior [6]. Although preclinical studies have shown that maladaptive memories can be weakened or eliminated by drugs or behavioral manipulation during reconsolidation, the results of transformational clinical studies are inconsistent [7]. In addition, due to the complexity of most human memories, the molecular mechanism underlying memory reconsolidation has not been fully understood [8]. Therefore, a better understanding of the process and molecular mechanism of memory reconsolidation is critical for the treatment of psychiatric diseases characterized by maladaptive memories.

N6-methyladenosine (m6A) is the most abundant dynamic mRNA modification in the brain [9]. With its role in regulating mRNA processing, m6A modification has become an important and multifunctional regulator in the function of the CNS, including neural plasticity, learning and memory [9]. In particular, altered expression levels of the m6A regulators including Fat mass and obesity associated gene (FTO), ELAVL1, and YTHDF2 were found in Alzheimer’s disease and were related to pathological development and cognitive levels [10]. FTO is the first discovered mRNA m6A demethylase, which is critical for mRNA alternative splicing and translation [11]. FTO has also been involved in the pathogenesis of mood disorders, suggesting its crucial function in the CNS [12, 13]. At present, several lines of evidence have shown that FTO plays a role in learning and memory. Interestingly, a study found that the down-regulation of m6A eraser FTO and the resulting increase in global m6A methylation levels were also associated with learning and memory [13]. Conditionally knocked out FTO not only decreased the level of lipids in brain tissue, but also impaired the learning and memory ability of mice [14]. Deletion of FTO in adult neurons altered the coding group of m6A/m epitopes, increased fear memory, and changed the response of transcriptome to fear and synaptic plasticity [15]. More recently, it was shown that FTO knockout results in abnormal reward learning and FTO is necessary to repair neurological damage after traumatic brain injury (TBI) [16–18]. Although the regulatory effect of FTO on learning and memory has been well studied, the functional role of FTO in memory reconsolidation has not been examined.

Brain-derived neurotrophic factor (BDNF) is a key member of the neurotrophic family and highly enriched in the hippocampal and cortical neurons. In addition to the well-documented roles in neuronal proliferation and survival, BDNF has been extensively involved in regulating learning and memory *via* its high-affinity receptor tyrosine kinase receptor B (TrkB) [19]. An essential role of BDNF in memory reconsolidation has been documented in the novel object recognition (NOR) model [20]. Additionally, ketamine promoted NOR memory reconsolidation through the BDNF-TrkB pathway [21]. Interestingly, it has been reported that FTO deficiency downregulated hippocampal BDNF, suggesting a regulatory effects of FTO on the BDNF-TrkB pathway [22]. However, whether the FTO-mediated BDNF-TrkB pathway is involved in memory reconsolidation has not been investigated.

In the present study, we hypothesized that FTO participated in memory reconsolidation through the BDNF-TrkB pathway. To test this hypothesis, the role of hippocampal FTO in memory reconsolidation and the mechanism associated with the BDNF-TrkB pathway will be investigated in a NOR model in mice. To further explore the m6A-dependent mechanism, the effects of FTO on the BDNF mRNA m6A levels will be investigated in the HT22 cells.

## Materials and methods

### Animals

Male C57BL/6J mice (7w, weighing 20 to 25 g) were purchased from SJA Company, Changsha, China. They were housed in a climate-controlled vivarium with 12 h day-night cycle (the light is on at 8:00-20:00). The laboratory temperature is maintained at 22–25 °C and the relative humidity is 40–60%. All trials were conducted in accordance by the National regulations on the Health and Management of Experimental Animals issued by the National Institutes of Health (NIH) and were approved by the Animal Ethics Committee of the University of South China (No. 2023-325).

### Novel object recognition (NOR)

The NOR experiments was performed as previously described with minor modifications [21]. Specifically, the NOR experiments was carried in an open field box with a length, width, and height of 40 × 40 × 40 cm. The experimental process includes adaptation, training, activation, and detection stage: (1) adaptation: no objects are placed in the open field box, and the mice are put into the open field box in turn and are allowed to move freely for 10 min; (2) training: two identical objects AA are placed in the open field box and then the mice are put into the open field box in turn and allowed to move freely for 10 min; (3) on the day after training, a familiar object A in the open field box was replaced with a novel B object, and the mice were put into the box and allowed to move freely for 10 min to reactivate the memory of object A. After activation, different drugs were injected as described below; (4) detection: on the day after activation, object B was replaced with a new object C, and the mice were put into the open field box again, and the times of exploration of object A and C were recorded. The exploration was defined when the mice was sniffing or touching the stimuli objects with the muzzle and/or forepaws [21]. Two investigators blinded to groups were in charge of making the statistics. The memory of object A (old object) was measured by discrimination index (DI), which was calculated as DI = (time of novel object explorations-time of old object explorations)/total time of explorations.

### Drug intervention

#### Meclofenamic acid (MA) treatment

**Fig. 1:**
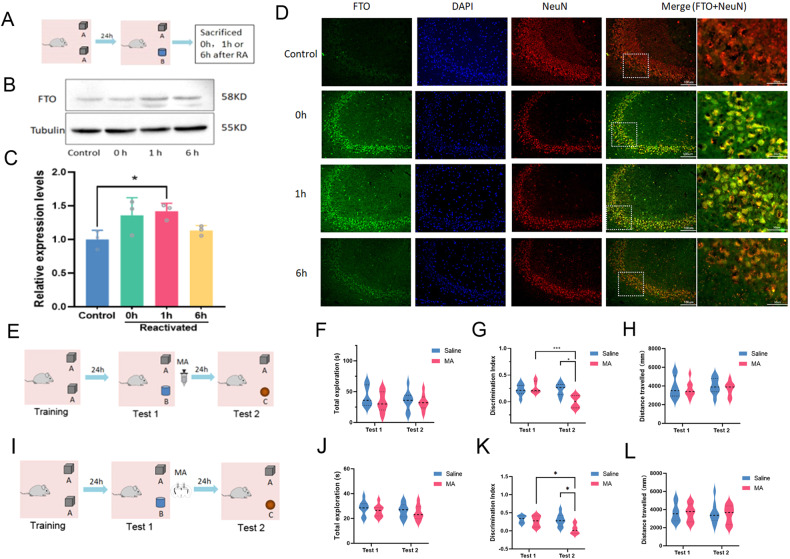
The essential role of FTO in NOR memory reconsolidation. **A** Schematic of the experimental design for NOR memory RA and sampling. **B** Representative blots of FTO. **C** Quantitative analysis of FTO expression in WB. *n* = 3. *P = 0.041. **D** Representative IFC images of FTO and NeuN double-staining in the hippocampus CA3 area. Scale bar, 100 μm (left 4 columns), 50 μm (right column). **E** Schematic of the experimental design for the effects of MA, a FTO inhibitor, administered immediately after RA on NOR memory performance (*n* = 8). **F** Total exploration times in Test 1 and Test 2. **G** Discrimination index (DI) of each group in Test 1 and Test 2. ***P = 0.0051, *P = 0.0110. **H** Distance of travelled during the tests. **I** Schematic of the experimental design for the effects of hippocampal infusion of MA immediately after RA on NOR memory performance (*n* = 8). **J** Total exploration times in Test 1 and Test 2. **K** Discrimination index (DI) of each group in Test 1 and Test 2. *P = 0.0089, *P = 0.047. **L** Distance of travelled during the tests.

Mice were randomly divided into Sal group and MA group. In the Sal group, saline was injected intraperitoneally (IP) after no activation or reactivation, and the second test was performed 24 h later. While in the MA group, 5 mg/kg of MA (an FTO inhibitor) (M4531, Sigma) [23] was injected (I.P.) immediately or 6 h after reactivation or no reactivation (Fig. 1E). The DI of familiar object A and new object C was used to evaluate the memory to object A. To infuse MA to the hippocampal CA1 region, a group of mice (*n* = 16) we bilaterally implanted with 30-gauge cannula (RWD life science, Shenzhen, China) using a stereotaxic apparatus using the coordinates (anteroposterior, −2.0 mm; mediolateral, ±1.5 mm; dorsoventral, −1.7 mm) under the anesthesia of isoflurane and was secured by dental cement [23, 24]. After recovery from the surgery for 7 days, the mice were subjected to NOR training and reactivation. Immediately after the reactivation of NOR memory, Saline or MA (1 mM) (2 μL in 3 min) was infused to the hippocampal CA1 region of the mice through the cannula [23]. On the other day, the mice were tested for NOR memory again as described above (Fig. 1I).

#### ANA-12treatment

**Fig. 2:**
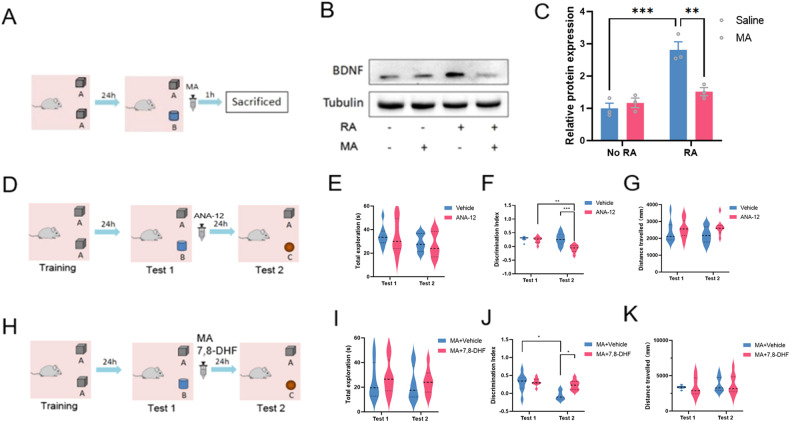
FTO mediated the BDNF-TrkB pathway in NOR memory reconsolidation. **A** Schematic of the experimental design for NOR memory RA and sampling. **B** Representative BDNF blots of each group. **C** Quantitative analysis of BDNF expression in WB. *N* = 3. ***P = 0.0004, **P = 0.0036. **D** Schematic of the experimental design for the effects of ANA-12, a TrkB antagonist, administered immediately after RA on NOR memory performance. **E** Total exploration times in Test 1 and Test 2. **F** DI of each group in Test 1 and Test 2. **P = 0.0013, ***P = 0.0006. **G** Distance of travelled during the tests. **H** Schematic of the experimental design for the effects of 7,8-dihydroxyflavone (7,8-DHF), a TrkB agonist, on MA mediated disruption of NOR memory reconsolidation. **I** Total exploration times in Test 1 and Test 2. **J** DI of each group in Test 1 and Test 2. *P = 0.0287, DI of vehicle + MA group between Test 1 and Test 2; *P = 0.0216, DI between vehicle + MA group and vehicle + 7,8-DHF group in Test 2. **K** Distance of travelled during the tests.

Mice were randomly divided into Vehicle group and ANA-12 group. The mice in the Vehicle group were injected with the Vehicle (DMSO+Saline) immediately after reactivation, and the second test was performed 24 h later, while the mice in the ANA-12 group received IP injection of 0.5 mg/kg ANA-12 (a TrkB inhibitor) (SML0209, Sigma) [25] immediately after reactivation (Fig. 2D). The DI of familiar object A and new object C was used to evaluate the memory ability of mice to object A.

#### 7,8-dihydroxyflavone(7,8-DHF) treatment

The mice were randomly divided into MA+Vehicle group and MA + 7,8-DHF group. In MA+Vehicle group, MA (5 mg/kg) was injected IP immediately after RA, and the equal volume of Vehicle (DMSO + H_2_O) was injected 10 min later. In MA + 7,8-DHF group, MA (5 mg/kg) was injected IP immediately after RA, and 5 mg/kg 7,8-DHF (a TrkB agonist) (D5446, Sigma) [26] was injected IP 10 min later. Mice of both groups were detected with A and C objects again after 24 h.

### Cell culture and treatment

HT22 mouse hippocampal neuronal cell line was purchased from iCell Bioscience Inc, Shanghai China. Cells were cultured in a cell culture flask containing Dulbecco’s modified Eagle’s medium (DMEM) (8122542, Thermo Fisher Scientific, China) with 10% fetal bovine serum (G8001, Servicebio, Wuhan, China) in a humidified incubator at 37 °C with 5% CO_2_. The culture medium was changed every 2–3 days. When the cells reached 90% confluence, they were detached by 0.25% trypsin (G4005, Servicebio, Wuhan, China) and transferred to a 6-well plate for the subsequent experiments or passaged in a new flask. For the MA treatment, 10 mmol/L MA (M4531-1 Sigma, USA) was added to the cell culture medium; for the control group, the equivalent volume of saline was added to the cell culture medium. The cells were collected 24 h after the treatment for further western blotting or PCR analysis. For the FTO overexpression experiment, Lentivirus system (pLenti-EF1-mCherry-P2A-Neo- CMV-Fto-3/FLAG-WPRE, 4.64 × 10^8^ TU/mL) was constructed by Obio Technology (Shanghai) Corp., Ltd., China and the pLenti-EF1-mCherry-P2A-Neo-CMV- MCS-3/FLAG-WPRE (3.37 × 10^8^ TU/m L) was used as control. The transfection efficiency was confirmed by a fluorescence microscope. The cells were collected 48 h after the transfection for further western blotting or PCR analysis.

### Western blotting (WB)

For the hippocampal tissue analysis, mice were euthanized under isoflurane anesthesia after the behavioral test and the brains were rapidly removed on ice and stored at −80 °C. For the cultured cell sample, the cells were lysed after the experiments and stored at −80 °C for follow-up experiments. Before the western blotting experiments, the frozen hippocampal tissues or cell culture sample were homogenized, and lysed in RIPA buffer with protease inhibitor. BCA protein concentration detection kit was used to determine protein concentration (R0020, Solarbio, Beijing, China). The protein samples were separated by SDS-PAGE electrophoresis for 90 min at 120 V, and transferred to nitrocellulose membrane by 90 min at 90 V constant pressure, blocked with 5% skimmed milk (dissolved in PBS containing Tween 20) at room temperature for 2 h, and incubated overnight with corresponding antibodies at 4 °C: Tublin (1:2000, 66240-1-Ig, Proteintech, Wuhan, China), or Actin (1:2000, 66009-1-Ig Proteintech, Wuhan, China), FTO (1:500, 27226-1-AP, Boster Biological Technology, Wuhan, China), or BDNF (1:400, BM4113, Boster Biological Technology, Wuhan, China), or TrkB (1:1000, 13129-1-AP, Proteintech, Wuhan, China). After washing with PBST for 3 times (3 × 10 min), membranes were incubated with horseradish peroxidase-conjugated goat anti-rabbit (1:2000, SA00001-2, Proteintech, Wuhan, China) or goat anti-mouse IgG1:2000, SA00001-1, Proteintech, Wuhan, China) at room temperature for 2 h. Then the band was developed with enhanced chemiluminescence (ECL) (CW0049M, ComWin Biotech. Beijing, China), and the scanned images were quantified using the Image J software.

### Immunofluorescence (IFC)

The expression of FTO in mouse hippocampal neurons was detected by IFC staining. 0 h, 1 h, and 6 h after RA of NOR memory, the mice were anesthetized with isoflurane and were perfused with 4% paraformaldehyde (PFA). The brains were removed further fixed with 4% PFA for 24 h, followed by dehydration with 10%, 20%, 30% sucrose solution for 24 h each. And the brains were sectioned in a Cryostat (Lecia 3050, Germany) at thickness of 15 μm. For IFC staining, the slides were washed 3 times with PBS and incubated with 0.1% Triton for 10 min, blocked with BSA (5%) for 2 h at room temperature. The slides were incubated overnight at 4 °C with the corresponding primary antibody for FTO (1:50, 27226-1-AP, Wuhan Boster Biological Technology Ltd., China) and NeuN (66836-1-Ig, Proteintech Group, Inc Wuhan, China). On the next day, the slides were recovered at room temperature for 1 h and washed with PBST 5 min for 3 times. The slides were incubating with the Alexa Fluor™ Plus 488-conjugated goat anti-rabbit antibody (1:2000, A32731, Invitrogen) and Alexa Fluor™ Plus 594-conjugated goat anti-mouse antibody (1:2000, A32742, Invitrogen) at room temperature 1 h. The slides were washed with PBST for 3 times and stained with DAPI (G1012, Servicebio, Wuhan, China) for 6 min and mounted with anti-fade mounting medium. The results were observed and photographed using a Nikon fluorescence microscope.

### Real-time PCR (RT-PCR) and quantitative detection of RNA m6A levels

After the treatment, HT22 cells were lysed with Trizol and RNA was extracted using TRIzol Reagent (15596026, Thermo Fisher Scientific, Shanghai, China). cDNA was synthesized according to the instructions of the reverse transcriptase kit (K1662, Thermo Fisher Scientific, Shanghai, China). PCR reaction system: 5 μL TB Green Premix, 0.2 μL Rox Reference Dye, 0.8 μL primer (GAPDH: *Forward*, ACC ACC ATG GAG AGG GCT GG; *Reverse*, CTC AGT GTA GCC CAG GAT GC; BDNF: *Forward*, GAC GACATC ACT GGC TGA CAC T; *Reverse*, CCG AAC CTT CTG GTC CTC ATC C), 3 μL enzyme free water, 1 μL cDNA. The amplification conditions of PCR were as follows: pre-denaturation at 95 °C for 30 s, denaturation at 95 °C for 5 s, annealing at 60 °C for 45 s, repeated denaturation and annealing for 40 cycles. The GAPDH gene was used as the internal reference. The relative expression of mRNA was calculated by 2^-delta delta Ct^ method.

The total RNA was subjected to m6A containing RNA enrichment using a MeRIP kit(D7283S, AmyJet Scientific, Wuhan, China) and the region-specific m6A was identified by polymerase chain reaction. The steps are as follows: 5 μg RNA was mixed with other reagents (ICB: 188.5 μL; m6A antibody: 2 μL; Affinity Beads: 4 μL) to form a total volume of 200 μL immune capture solution, which was added to a 200 μL enzyme-free EP tube; the EP tube was fixed on a shaker at room temperature for 90 min; then 10 μL NDE (Nuclear Digestion Enhancer) and 2 μL CEM (Cleavage Enzyme Mix) were added to each tube and incubated at room temperature for 4 min; the EP tube was placed on a magnetic device until the solution became clear (about 2 min), and the supernatant was discarded. After wash 3 times with WB (Wash Buffer) and PDB (Protein Digestion Buffer), 20 μL protein digestive juice was added (Proteinase K:PDB = 1:10) to each tube. After the tube were put in a water bath at 55 °C incubated for 15 min, the EP tube was put on the magnetic device again until the solution is clear and the solution to the were carefully move to a new EP tube; 20 μL’s RPS (RNA Purification Solution) and 160 μL of 100% anhydrous ethanol were added to each tube. Then 2 μL of RNA Binding Beads was added to each EP tube, turn it up and down for 10 times and incubate at room temperature for 5 min. It was put on a magnetic device until the solution is clear (about 2 min) and the supernatant was discarded. Elution Buffer (13 μL) was added and incubated at room temperature for 5 minutes and finally 13 μL m6A enriched RNA was transfer to a new EP tube. The products were analyzed for BDNF mRNA by qPCR as described above.

### Image analysis and statistical analysis

Image J software was used for picture processing and analysis. All counting data are expressed by Mean ± SEM. Prism8.0 software was used for statistical analysis. Unpaired t-test was used for two-group comparisons and one-way or two-way ANOVA was used for the comparisons of multiple groups. After the ANOVA, the Tukey’s post hoc test was used to compare the experimental data of each group. *P* < 0.05 was considered statistically significant.

## Results

### Hippocampal FTO is required for NOR memory reconsolidation

To determine whether FTO was involved in memory reconsolidation, we firstly investigated the effects of memory RA on hippocampal FTO expression. The mice were trained to recognize object A and the object memory was reactivated by exposure to object A and a novel object B for 10 min after 24 h of the training (Fig. 1A). The mice in the RA group were sacrificed at 0 h, 1 h or 6 h after the exposure respectively while the mice in the control group were sacrificed without exposure to any object (Fig. 1A). FTO expression was measured by WB and the representative blots of FTO were shown in Fig. 1B. One-way ANOVA showed that there was a main effect of memory RA on hippocampal FTO expression (F_(3, 8)_ = 4.292, *P* = 0.0441). Tukey’s post-hoc analysis showed that FTO expression was significantly increased in the hippocampus 1 h after memory RA as compared with the control (*P* = 0.041) (Fig. 1C). To further confirm the neuronal expression of FTO in the hippocampus, double staining of FTO and NeuN was performed after memory RA. As shown in Fig. 1D, the neuronal expression of FTO was significantly increased after memory RA but it was decreased 6 h after memory RA (Fig. 1D). These results demonstrated that hippocampal FTO expression was changed in a time-dependent manner after memory RA, suggesting a potential role of FTO in memory reconsolidation.

To determine whether FTO is required for NOR memory reconsolidation, we investigated the effects of MA, an FTO inhibitor, on NOR performance in mice (Fig. 1E–H). Immediately after the NOR memory reactivation (Test 1), the mice were injected with saline or MA (5 mg/kg). The discrimination index (DI) between object A and a novel object C was used to evaluate the memory performance of object A (Test 2) (Fig. 1E). Two-way ANOVA revealed no significant difference in the total exploration times (F_(1, 7)_ = 1.740, *P* = 0.2286), indicating the object exploring behavior was not altered among these groups. However, two-way ANOVA revealed that there was a significant difference in DI among these groups (F_(1, 7)_ = 15.03, *P* = 0.0061) (Fig. 1F). Tukey’s post-hoc analysis showed that the DI of MA treated group was significantly decreased in Test 2 as compared with Test 1 (*P* = 0.0051) (Fig. 1G). Tukey’s post-hoc analysis also showed the DI of MA treated group was significantly decreased as compared with the saline group in Test 2 (*P* = 0.0110) (Fig. 1G). Moreover, Two-way ANOVA revealed no significant difference in the total distance travelled among these groups (F_(1, 7)_ = 0.04519, *P* = 0.8377) (Fig. 1H), suggesting the difference in DI may not be attributed to the locomotor activity. The decreased DI in the MA-treated group indicated that MA treatment after memory RA may disrupt NOR memory reconsolidation.

Previous observed decreased DI may be also induced by MA itself instead of its inhibitive effects on memory reconsolidation. To exclude this, we further investigated the effects of MA 6 h after RA or without RA on NOR memory performance (Fig. S1A–D). When MA was IP injected 6 h after RA (Fig. S1A), two-way ANOVA revealed no significant difference in the total exploration times (F_(1, 7_) = 0.07229, *P* = 0.7958) (Fig. S1B), DI (F_(1, 7)_ = 0.1723, *P* = 0.6905) (Fig. S1C) and distance travelled (F _(1, 7)_ = 0.04519, *P* = 0.8377) (Fig. S1D), indicating that MA injection 6 h after RA did not affect the NOR memory. When MA was administered without a RA session (Fig. S2A), two-tailed t-test revealed no significant difference in the total exploration times (t = 1.088, *P* = 0.4992) (Fig. S2B), DI (t = 0.1124, *P* = 0.2929) (Fig. S2C) and distance travelled (t = 1.385, *P* = 0.7725) (Fig. S2D), indicating that MA injection without RA did not affect the NOR memory. These results indicated that MA alone did not affect the memory performance.

To further investigate whether the function of hippocampal FTO is required for NOR memory reconsolidation, Saline or MA(1 mM) (2 μL in 3 min) was infused to the bilateral hippocampus of mice immediately after the NOR memory reactivation (Test 1). The discrimination index (DI) between object A and a novel object C was used to evaluate the memory performance of object A (Test 2) (Fig. 1I). Two-way ANOVA revealed no significant difference in the total exploration times (F_(1, 7)_ = 0.4293, *P* = 0.5333) (Fig. 1J), indicating the object exploring behavior was not altered among these groups. However, two-way ANOVA revealed that there was a significant difference in DI among these groups (F_(1, 7)_ = 12.86, *P* = 0.0089) (Fig. 1K). Tukey’s post-hoc analysis showed that the DI of MA treated group was significantly decreased in Test 2 as compared with Test 1 (*P* = 0.0417) (Fig. 1K). The DI of MA treated group was significantly decreased as compared with the saline group in Test 2 (*P* = 0.0205) (Fig. 1G). However, Two-way ANOVA revealed no significant difference in the total distance travelled among these groups (F_(1, 7)_ = 0.06690, *P* = 0.8033) (Fig. 1L). These findings demonstrated that hippocampal FTO is required for the NOR memory reconsolidation. Taken together, our results revealed that NOR memory RA increased neuronal FTO expression in mice hippocampus and the FTO inhibitor MA injected immediately after RA disrupted NOR memory but not 6 h after the RA or without a RA session, indicating the hippocampal FTO is required for NOR memory reconsolidation.

### FTO participates in NOR memory reconsolidation via BDNF-TrkB pathway

To investigate the mechanism of FTO in memory reconsolidation, we firstly determined the effects of MA on BDNF expression after NOR memory RA in the hippocampus (Fig. 2A–C). Immediately after the RA, mice were injected with MA or saline and were sacrificed 1 h after the injection (Fig. 2A). The representative blots of BDNF were shown in Fig. 2B and the quantitative analysis of BDNF expression were shown in Fig. 2C. Two-way ANOVA showed that there was a significant interaction effects of MA treatments and RA on BDNF expression (F_(1, 8)_ = 17.29, *P* = 0.0031) (Fig. 2C). Tukey’s post-hoc analysis showed that BDNF expression was significantly increased after RA (*P* = 0.0004) while it was significantly decreased in the MA treatment group as compared with the control (*P* = 0.0036) (Fig. 2C). These results indicated that the FTO inhibitor MA down-regulates BDNF expression during NOR memory reconsolidation in the hippocampus.

Previous studies have found that the BDNF antibody disrupted NOR memory reconsolidation, suggesting a critical role of BDNF in NOR memory reconsolidation. However, it is undetermined whether BDNF participated in NOR memory reconsolidation via its high-affinity receptor TrkB. Hence, we investigated the effects of TrkB inhibitor ANA-12 on NOR memory reconsolidation (Fig. 2D–G). Mice were trained to recognize object A and IP injected with ANA-12 (0.5 mg/kg) immediately after RA. The NOR memory performance was tested again on another day with the familiar object A and a novel object C (Fig. 2D). Two-way ANOVA revealed no significant difference in the total exploration times (F_(1, 7)_ = 0.09815, *P* = 0.7632) (Fig. 2E). However, two-way ANOVA revealed that there was a significant difference in DI among these groups (F_(1, 7)_ = 21.05, *P* = 0.0025) (Fig. 2F). Tukey’s post-hoc analysis showed that the DI of ANA-12 treated group was significantly decreased in Test 2 as compared with Test 1 (*P* = 0.0013) (Fig. 2F). Tukey’s post-hoc analysis also showed the DI of ANA-12 treated group was significantly decreased as compared with the vehicle group in Test 2 (Fig. 2F). Moreover, Two-way ANOVA revealed no significant difference in the total distance travelled among these groups (F_(1, 7)_ = 0.04519, *P* = 0.8377) (Fig. 2G). These results indicated that BDNF was involved in NOR memory reconsolidation through its high-affinity receptor TrkB.

Given the results that FTO inhibitor MA disrupted NOR memory reconsolidation and downregulated BDNF in the hippocampus, we further investigated the effects of 7,8-dihydroxyflavone (7,8-DHF), a TrkB agonist, on MA mediated disruption of NOR memory reconsolidation (Fig. 2H–K). Mice were trained to recognized object A and were injected with 7,8-DHF (5 mg/kg) or vehicle (DMSO) 15 min prior to the MA injection immediately after RA. The NOR memory performance was tested again on another day with the familiar object A and a novel object C (Fig. 2H). Two-way ANOVA revealed no significant difference in the total exploration times (F_(1, 7)_ = 0.01080 *P* = 0.9201) (Fig. 2I). However, two-way ANOVA revealed that there was a significant difference in DI among these groups (F_(1, 7)_ = 7.727, *P* = 0.0273) (Fig. 2J). Tukey’s post-hoc analysis showed that the DI of vehicle + MA treated group was significantly decreased in Test 2 as compared with Test 1 (*P* = 0.0287) (Fig. 2J). Tukey’s post-hoc analysis also showed the DI of 7,8-DHF + MA treated group was significantly increased as compared with the vehicle + MA group in Test 2 (Fig. 2J) (*P* = 0.0216). Moreover, Two-way ANOVA revealed no significant difference in the total distance travelled among these groups (F_(1, 7)_ = 0.03444, *P* = 0.8580) (Fig. 2K). Taken together, these results indicated that the disruptive effects of MA on NOR memory reconsolidation was reversed by 7,8-DHF, suggesting MA may inhibit NOR memory reconsolidation through down-regulating BDNF.

### FTO regulates the BDNF-TrkB pathway via m6A demethylation

**Fig. 3:**
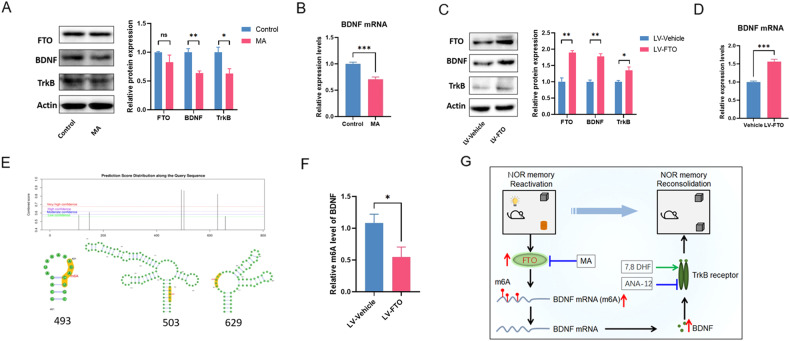
The m6A-dependent mechanism of FTO in regulating the BDNF-TrkB pathway. **A** Western blotting shows the relative protein expression levels of FTO, BDNF, and TrkB in Control and MA treated HT22 cells (*n* = 3); **P < 0.01, *P < 0.05. **B** RT-PCR shows the relative levels of BDNF mRNA in Control and MA treated HT22 cells (*n* = 3); ***P < 0.001. **C** Western blotting shows the relative protein expression levels of BDNF in LV-Vehicle and LV-FTO HT22 cells (*n* = 3). **P < 0.01, *P < 0.05. **D** RT-PCR shows the relative levels of BDNF mRNA in LV-Vehicle and LV-FTO HT22 cells (*n* = 3); ***P < 0.001. **E** Prediction of m6A modification site on BDNF mRNA. **F** RT-PCR shows the m6A modification levels of BDNF mRNA in LV-Vehicle and LV-FTO cells (*n* = 5). *P < 0.05. **G** Schematic of the role and mechanisms of FTO in NOR memory reconsolidation. The reactivation of NOR memory increased hippocampal FTO expression, which decrease the m6A level of BDNF mRNA and increased the protein expression of BDNF. BDNF binds to the high-affinity receptor TrkB to promote the NOR memory reconsolidation. The FTO inhibitor MA and TrkB antagonist ANA-12 disrupt NOR memory reconsolidation via targeting the FTO-BDNF-TrkB pathway.

In order to investigate whether FTO regulates the BDNF-TrkB signal pathway through m6A demethylation, we first treated the HT22 neurons cells with FTO inhibitor MA (10 mM, 24 h), and measured the protein expression using WB. Although the MA did not change the expression of FTO (*P* = 0.23603), we found significant down-regulation of BDNF (*P* = 0.00733) and TrkB (*P* = 0.03471) at the protein level after the MA treatment (Fig. 3A). To further explore the effects of MA on BDNF mRNA level, the RT-PCR was also performed after MA treatment in the cells and the results showed that BDNF mRNA in the MA group was significantly decreased (*P* = 0.0007) (Fig. 3B). These results indicated that the FTO inhibitor MA decrease BDNF-TrkB pathway via down-regulating BDNF mRNA levels. Next, FTO was overexpressed in HT22 cells with LV-FTO (*P* = 0.00233) (Fig. 3C). WB also showed that the expression of BDNF (*P* = 0.00128), and TrkB (*P* = 0.0343) was increased by FTO overexpression (Fig. 3C). In addition, RT-PCR showed that BDNF mRNA level was significantly increased in LV-FTO group (*P* = 0.0002) (Fig. 3D). These results indicated that FTO increased BDNF-TrkB pathway in HT22 cells. Taken together, these findings suggested that FTO played a crucial regulatory role in BDNF-TrkB pathway expression.

To further investigate the effects of m6A demethylation on BDNF expression at mRNA level, we used an online tool-SRAMP [27] to predict the m6A modification site on BDNF mRNA. As shown in Fig. 3E, three very-high confidence sites were found on BDNF mRNA. Furthermore, MeRIP-PCR showed overexpression of FTO significantly decreased m6A level of BDNF mRNA (*P* = 0.0329) (Fig. 3F). These results demonstrated that FTO regulates BDNF-TrkB signal pathway through m6A modification.

## Discussion

The main purpose of the study is to investigate the role of hippocampal FTO in NOR memory reconsolidation. NOR model is a relatively fast and effective method for testing different stages of learning and memory in rodents. Here, we used the NOR model to demonstrate that FTO is a novel molecule involved in memory reconsolidation in mice. We found that the expression of FTO in the hippocampus was increased significantly after NOR memory reactivation in a time-dependent manner, and FTO inhibitor MA could impair NOR memory reconsolidation in mice. Additionally, TrkB receptor agonist 7,8-DHF could reverse the inhibitory effect of MA on NOR memory reconsolidation in mice. Moreover, MA decreased BNDF expression, and overexpression of FTO increased BDNF expression associated with decreased mRNA m6A levels in HT22 cells. These results indicate that hippocampal FTO participated in NOR memory reconsolidation through the BDNF-TrkB pathway.

FTO is a highly abundant RNA demethylase in the brain and is highly expressed in adult neural stem cells (ANSCs) [28]. FTO has been found to be related to memory consolidation and participate in the regulation of dopaminergic brain network [29, 30]. In another study, Brandon et al found that specifically reducing the level of FTO in hippocampal neurons can enhance episodic fear memory and found that it is associated with an increase in the level of methylated mRNA [31]. These findings suggested that FTO plays an important role in learning and memory, but the role of FTO in memory reconsolidation has not been reported. In order to determine whether FTO is involved in the reconsolidation process of NOR memory in mice, we first detected the expression of FTO in hippocampal CA3 neurons after activating NOR memory by IFC. We found that the expression of FTO changed time dependently in the hippocampus after reactivation of NOR memory in mice. Moreover, the expression of FTO in hippocampus of activated group (1 h group) was significantly higher than that of inactivated group (1 h group), suggesting FTO may be a key molecule regulating the reconsolidation of NOR memory.

MA is a highly selective inhibitor of FTO and it can effectively and selectively inhibit FTO demethylation by competing with substrates containing m6A [32]. In order to further verify whether FTO is required for the process of NOR memory reconsolidation, we first observed the effect of FTO inhibitor MA on NOR memory reconsolidation in mice. Previous studies have found that MA inhibits FTO demethylation of single-stranded DNA or single-stranded RNA containing m6A in a dose-dependent manner within 2 h at room temperature [32]. Through the pilot study, we found an effective inhibitory dosage of MA at 5 mg/kg, which is consistent with the previous study [33]. After reactivating the memory of electric shock, protein synthesis inhibitor (PSI) injected into the basolateral amygdala only disrupts fear memory within 6 h, indicated there was a time limit for memory reconsolidation [34]. Moreover, Schiller et al. also found that treatment between 10 min to 6 h after reactivation could enhance fear conditioning extinction [35]. In this study, the mice were injected with FTO inhibitor MA immediately or 6 h after memory activation, and the memory to object A between familiar object A and new object C by using the DI. It was found that only MA can disrupt NOR memory reconsolidation immediately after memory reactivation, but not 6 h after reactivation or without no reactivation in mice, suggesting FTO is required for the reconsolidation of NOR memory in mice.

It has been reported that FTO affects the function of the hippocampus by regulating the expression of BDNF, and found that the level of BDNF in the hippocampus of FTO knockout mice is significantly decreased [22], suggesting that BDNF may be an important molecular target of FTO. Therefore, we explored whether FTO participates in NOR memory reconsolidation through BDNF. As early as 2012, Samartgis et al. found that BDNF can promote memory consolidation and reconsolidation of weak training stimulation in chicken using the passive avoidance learning paradigm, indicating that BDNF is necessary for memory reconsolidation for the first time [36]. Therefore, after activating NOR memory in mice, we injected the mice with MA, an inhibitor of FTO, and detected the expression of BDNF in the hippocampus of mice by WB. Our results showed that the expression of BDNF during NOR memory reconsolidation was significantly decreased after MA injection, which is consistent with the previous study [37]. BDNF plays a crucial role in learning and memory mainly by binding to its high-affinity receptor TrkB [38]. However, it is uncertain whether BDNF participates in NOR memory reconsolidation through the TrkB receptor. Therefore, we explored the effect of TrkB inhibitor ANA-12 (I.P. injection 0.5 mg/kg) on memory reconsolidation in NOR. The results showed that ANA-12 destroyed the reconsolidation of NOR memory in mice, indicating that BDNF participates in the reconsolidation of NOR memory through the TrkB receptor.

Nextly, we explored the influence of FTO on the BDNF-TrkB signal pathway. As a result of the above studies, we have confirmed that FTO inhibitor MA can disrupt the process of reconsolidation of NOR memory in mice. Therefore, we then employed a TrkB receptor agonist 7,8-DHF to see if it could reverse the destructive effect caused by MA. 7,8-DHF is a member of the flavonoid family, which is similar to BDNF and has a high binding affinity to the TrkB receptor. Because it can cross the blood-brain barrier and has safe pharmacokinetic characteristics, 7,8-DHF has become a commonly used agent against neuropsychiatric disorders [39]. After reactivation of NOR memory, we injected the mice with 7,8-DHF (5 mg/kg) 10 min after MA injection and the results showed that 7,8-DHF could reverse the disruptive effect of MA on NOR memory reconsolidation in mice. Hence, our existing results suggest that FTO may play a role in NOR memory reconsolidation by up-regulating the BDNF-TrkB signal pathway.

As an m6A demethylase, FTO can decrease the level of endogenous m6A on mRNA [11] and it was also found that FTO regulates the expression of BDNF-TrkB in the hippocampus through m6A-dependent post-transcriptional regulation, and participates in cognitive behavior impairment in mice under continuous light exposure [40]. In addition, it was also found that the mRNA level of BDNF was significantly increased in cells by FTO overexpression [41]. Based on these results, we further explored the potential effects of FTO on BDNF expression and mRNA m6A in HT22 cells [42]. We found that FTO inhibitor MA significantly downregulated BDNF at both mRNA and protein levels TrkB were downregulated after the treatment of MA. Then, in order to further verify the relationship between FTO and BDNF, we overexpressed FTO in HT22 cells and found that BDNF was increased significantly at both mRNA and protein level, and TrkB was also increased significantly. These results confirmed a regulatory role of FTO on the BDNF-TrkB pathway.

Previously, it was reported that the knockout of FTO in HeLa cells increased mRNA m6A levels [32]. Therefore, we speculate that FTO may up-regulate the expression of BDNF by removing m6A modification on the BDNF mRNA. In order to further verify this hypothesis, we first predicted the m6A modification sites using SRAMP [27] and found there are three high probability m6A modification sites on BDNF mRNA. To further confirm the BDNF m6A modification by FTO, MeRIP-PCR experiment was carried out and the results showed that the m6A level of BDNF mRNA decreased significantly after overexpression of FTO. Previous studies have confirmed that the ubiquitous m6A modification plays a basic regulatory role in gene expression [43, 44], and the dynamic modification of m6A is recognized by selective binding to proteins, which further affects the translation state and degradation of mRNA. Because mRNA m6A modification may up-regulate its expression by promoting mRNA translation, or inhibit protein expression by promoting mRNA degradation [45]. Therefore, we speculate that FTO may up-regulate the expression of BDNF protein by reducing the modification of BDNF mRNA m6A and promoting the stability of mRNA.

There are also several limitations in the present study. First, several brain regions may participate in the learning and memory process and we only paid attention to the hippocampus because our pilot studies showed that reactivation of NOR memory increased neuronal activity in the hippocampus but not in the prefrontal cortex. Although we performed the systematic injection (I.P.) and the stereotaxic infusion of the FOT inhibitor MA to the hippocampus, involvement of the other brain regions in NOR memory reconsolidation still cannot be ruled out. Second, because we only examined the m6A modification on the BDNF mRNA but not on the TrkB mRNA, it is possible that FTO may also affect the expression of TrkB directly. Last but not the least, we only tested the role and mechanism of FTO in the NOR model, whether FTO also mediated memory reconsolidation in other paradigms such as drug addiction and fear conditioning merits further investigations.

In summary, our results demonstrated a previously unclarified role of FTO in memory reconsolidation and revealed the mechanism underlying hippocampal FTO participating in NOR memory reconsolidation through up-regulating the BDNF-TrkB signal pathway. Further studies in hippocampal neurons HT22 cells found that FTO may activate the BDNF-TrkB signal pathway by reducing the m6A modification of BDNF mRNA and up-regulating its expression (Fig. 3G). These results not only clarify a novel role of FTO in memory reconsolidation but also reveal the possible molecular mechanism by which FTO participates in NOR reconsolidation through regulating BDNF, which provides a new molecular target for regulating and updating memory by intervening in the process of memory reconsolidation.

### Supplementary information

Fig S1

Fig S2

## Data Availability

The data supporting the findings of this study are available from the corresponding author upon reasonable request.

## References

[1] Lee JLC, Nader K, Schiller D (2017). An update on memory reconsolidation updating. Trends Cogn Sci.

[2] Liu X, Ma L, Li HH, Huang B, Li YX, Tao YZ (2015). β-Arrestin-biased signaling mediates memory reconsolidation. Proc Natl Acad Sci USA.

[3] Raut SB, Marathe PA, van Eijk L, Eri R, Ravindran M, Benedek DM (2022). Diverse therapeutic developments for post-traumatic stress disorder (PTSD) indicate common mechanisms of memory modulation. Pharm Ther.

[4] Taujanskaitė U, Cahill EN, Milton AL (2021). Targeting drug memory reconsolidation: a neural analysis. Curr Opin Pharm.

[5] Elharrar E, Dikshtein Y, Meninger-Mordechay S, Lichtenstein Y, Yadid G (2021). Modulation of PARP-1 activity in a broad time window attenuates memorizing fear. Int J Mol Sci.

[6] Leite Junior JB, de Mello Bastos JM, Samuels RI, Carey RJ, Carrera MP (2019). Reversal of morphine conditioned behavior by an anti-dopaminergic post-trial drug treatment during re-consolidation. Behav Brain Res.

[7] Astill Wright L, Horstmann L, Holmes EA, Bisson JI (2021). Consolidation/reconsolidation therapies for the prevention and treatment of PTSD and re-experiencing: a systematic review and meta-analysis. Transl Psychiatry.

[8] Jardine KH, Huff AE, Wideman CE, McGraw SD, Winters BD (2022). The evidence for and against reactivation-induced memory updating in humans and nonhuman animals. Neurosci Biobehav Rev.

[9] Han M, Liu Z, Xu Y, Liu X, Wang D, Li F (2020). Abnormality of m6A mRNA methylation is involved in Alzheimer’s disease. Front Neurosci.

[10] Liu Z, Xia Q, Zhao X, Zheng F, Xiao J, Ge F (2023). The landscape of m6A regulators in multiple brain regions of Alzheimer’s disease. Mol Neurobiol.

[11] Jia G, Fu Y, Zhao X, Dai Q, Zheng G, Yang Y (2011). N6-methyladenosine in nuclear RNA is a major substrate of the obesity-associated FTO. Nat Chem Biol.

[12] Liu S, Xiu J, Zhu C, Meng K, Li C, Han R (2021). Fat mass and obesity-associated protein regulates RNA methylation associated with depression-like behavior in mice. Nat Commun.

[13] Mitsuhashi H, Nagy C (2023). Potential roles of m6A and FTO in synaptic connectivity and major depressive disorder. Int J Mol Sci.

[14] Engel M, Eggert C, Kaplick PM, Eder M, Röh S, Tietze L (2018). The role of m6A/m-RNA methylation in stress response regulation. Neuron.

[15] Gao H, Cheng X, Chen J, Ji C, Guo H, Qu W (2020). Fto-modulated lipid niche regulates adult neurogenesis through modulating adenosine metabolism. Hum Mol Genet.

[16] Leonetti AM, Chu MY, Ramnaraign FO, Holm S, Walters BJ (2020). An emerging role of m6A in memory: a case for translational priming. Int J Mol Sci.

[17] Widagdo J, Wong JJ-L, Anggono V (2022). The m6A-epitranscriptome in brain plasticity, learning and memory. Semin Cell Dev Biol.

[18] Yu J, Zhang Y, Ma H, Zeng R, Liu R, Wang P (2020). Epitranscriptomic profiling of N6-methyladenosine-related RNA methylation in rat cerebral cortex following traumatic brain injury. Mol Brain.

[19] Palasz E, Wysocka A, Gasiorowska A, Chalimoniuk M, Niewiadomski W, Niewiadomska G (2020). BDNF as a promising therapeutic agent in Parkinson’s disease. Int J Mol Sci.

[20] Fan J-F, Tang Z-H, Wang S-Y, Lei S, Zhang B, Tian S-W (2021). Ketamine enhances novel object recognition memory reconsolidation via the BDNF/TrkB pathway in mice. Physiol Behav.

[21] Radiske A, Rossato JI, Gonzalez MC, Köhler CA, Bevilaqua LR, Cammarota M (2017). BDNF controls object recognition memory reconsolidation. Neurobiol Learn Mem.

[22] Spychala A, Rüther U (2019). FTO affects hippocampal function by regulation of BDNF processing. PLoS One.

[23] Jin M, Dai Y, Xu C, Wang Y, Wang S, Chen Z (2013). Effects of meclofenamic acid on limbic epileptogenesis in mice kindling models. Neurosci Lett.

[24] Tan S, Xue S, Behnood-Rod A, Chellian R, Wilson R, Knight P (2019). Sex differences in the reward deficit and somatic signs associated with precipitated nicotine withdrawal in rats. Neuropharmacology.

[25] Huang Z, Chang R, Peng J, Chen Y, Liao H, Zhao S (2022). Chronic nicotine up-regulates hippocampal BDNF-TrkB signaling pathway and ANA-12 inhibits nicotine conditioned place preference in mice. Asian J Psychiatr.

[26] Wang J, Gao F, Cui S, Yang S, Gao F, Wang X (2022). Utility of 7,8-dihydroxyflavone in preventing astrocytic and synaptic deficits in the hippocampus elicited by PTSD. Pharm Res.

[27] Zhou Y, Zeng P, Li Y-H, Zhang Z, Cui Q (2016). SRAMP: prediction of mammalian N6-methyladenosine (m6A) sites based on sequence-derived features. Nucleic Acids Res.

[28] Cao Y, Zhuang Y, Chen J, Xu W, Shou Y, Huang X (2020). Dynamic effects of Fto in regulating the proliferation and differentiation of adult neural stem cells of mice. Hum Mol Genet.

[29] Hess ME, Hess S, Meyer KD, Verhagen LAW, Koch L, Brönneke HS (2013). The fat mass and obesity associated gene (Fto) regulates activity of the dopaminergic midbrain circuitry. Nat Neurosci.

[30] Widagdo J, Zhao Q-Y, Kempen M-J, Tan MC, Ratnu VS, Wei W (2016). Experience-dependent accumulation of N6-methyladenosine in the prefrontal cortex is associated with memory processes in mice. J Neurosci.

[31] Walters BJ, Mercaldo V, Gillon CJ, Yip M, Neve RL, Boyce FM (2017). The role of The RNA demethylase FTO (fat mass and obesity-associated) and mRNA methylation in hippocampal memory formation. Neuropsychopharmacology.

[32] Huang Y, Yan J, Li Q, Li J, Gong S, Zhou H (2015). Meclofenamic acid selectively inhibits FTO demethylation of m6A over ALKBH5. Nucleic Acids Res.

[33] Soriano-Hernández AD, Galvan-Salazar HR, Montes-Galindo DA, Rodriguez-Hernandez A, Martinez-Martinez R, Guzman-Esquivel J (2012). Antitumor effect of meclofenamic acid on human androgen-independent prostate cancer: a preclinical evaluation. Int Urol Nephrol.

[34] Nader K, Schafe GE, Le Doux JE (2000). Fear memories require protein synthesis in the amygdala for reconsolidation after retrieval. Nature.

[35] Schiller D, Monfils M-H, Raio CM, Johnson DC, Ledoux JE, Phelps EA (2010). Preventing the return of fear in humans using reconsolidation update mechanisms. Nature.

[36] Samartgis JR, Schachte L, Hazi A, Crowe SF (2012). Brain-derived neurotrophic factor facilitates memory consolidation and reconsolidation of a weak training stimulus in the day-old chick. Neurosci Lett.

[37] Li L, Zang L, Zhang F, Chen J, Shen H, Shu L (2017). Fat mass and obesity-associated (FTO) protein regulates adult neurogenesis. Hum Mol Genet.

[38] Sasi M, Vignoli B, Canossa M, Blum R (2017). Neurobiology of local and intercellular BDNF signaling. Pflug Arch.

[39] Yang S, Zhu G (2022). 7,8-Dihydroxyflavone and neuropsychiatric disorders: a translational perspective from the mechanism to drug development. Curr Neuropharmacol.

[40] Yang Y, Feng Y, Hu Y, Liu J, Shi H, Zhao R (2021). Exposure to constant light impairs cognition with FTO inhibition and m6A-dependent TrκB repression in mouse hippocampus. Environ Pollut.

[41] Lin L, Hales CM, Garber K, Jin P (2014). Fat mass and obesity-associated (FTO) protein interacts with CaMKII and modulates the activity of CREB signaling pathway. Hum Mol Genet.

[42] Wang C, Cai X, Hu W, Li Z, Kong F, Chen X (2019). Investigation of the neuroprotective effects of crocin via antioxidant activities in HT22 cells and in mice with Alzheimer’s disease. Int J Mol Med.

[43] Dominissini D, Moshitch-Moshkovitz S, Schwartz S, Salmon-Divon M, Ungar L, Osenberg S (2012). Topology of the human and mouse m6A RNA methylomes revealed by m6A-seq. Nature.

[44] Meyer KD, Saletore Y, Zumbo P, Elemento O, Mason CE, Jaffrey SR (2012). Comprehensive analysis of mRNA methylation reveals enrichment in 3’ UTRs and near stop codons. Cell.

[45] Wang X, Lu Z, Gomez A, Hon GC, Yue Y, Han D (2014). N6-methyladenosine-dependent regulation of messenger RNA stability. Nature.

