# Supplementary material for: The hippocampal FTO-BDNF-TrkB pathway is required for novel object recognition memory reconsolidation in mice
Source: Transl Psychiatry. 2023 Nov 14;13:349. doi: 10.1038/s41398-023-02647-4 (PMC10645923; doi:10.1038/s41398-023-02647-4)

**Figure S1. A.** Schematic of the experimental design for the effects of MA, a FTO inhibitor, administered 6h after RA on memory performance (n=8). **B.** Total exploration times in Test 1 and Test 2. **C.** DI of each group in Test 1 and Test 2. **D.** Distance of travelled during the tests.


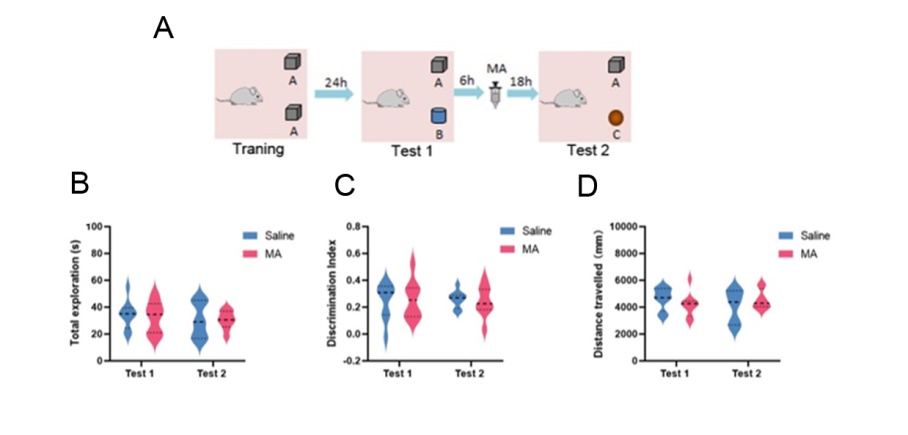

Supplement: Supplementary file 1 — Fig S1 [file 41398_2023_2647_MOESM1_ESM.docx]
